# An Innovative Concept for a Multivariate Plausibility Assessment of Simultaneously Recorded Data

**DOI:** 10.3390/ani10081412

**Published:** 2020-08-13

**Authors:** André Mensching, Marleen Zschiesche, Jürgen Hummel, Armin Otto Schmitt, Clément Grelet, Ahmad Reza Sharifi

**Affiliations:** 1 Animal Breeding and Genetics Group, Department of Animal Sciences, University of Goettingen, Albrecht-Thaer-Weg 3, 37075 Goettingen, Germany; rsharif@gwdg.de; 2 Center for Integrated Breeding Research (CiBreed), University of Goettingen, Albrecht-Thaer-Weg 3, 37075 Goettingen, Germany; armin.schmitt@uni-goettingen.de; 3 Ruminant Nutrition Group, Department of Animal Sciences, University of Goettingen, Kellnerweg 6, 37077 Goettingen, Germany; marleen.zschiesche@uni-goettingen.de (M.Z.); jhummel@gwdg.de (J.H.); 4 Breeding Informatics Group, Department of Animal Sciences, University of Goettingen, Margarethe von Wrangell-Weg 7, 37075 Goettingen, Germany; 5 Walloon Agricultural Research Center, Valorisation of Agricultural Products Department, Chaussée de Namur 24, 5030 Gembloux, Belgium; c.grelet@cra.wallonie.be

**Keywords:** multilevel data, plausibility assessment, sensor-based data acquisition

## Abstract

**Simple Summary:**

Benefiting from technical progress, it is nowadays easy to collect huge amounts of data using computerized sensor-based acquisition systems for both research and practical applications. However, such data often contain technology-related errors that are difficult to distinguish from physiologically extreme observations and thus can impair the quality of the data and also the statistical analysis. To tackle this, an innovative procedure for a multivariate plausibility assessment was developed to discriminate observations of simultaneously recorded traits between ‘physiologically normal’, ‘physiologically extreme’ or ‘implausible’ cases. To evaluate the performance and applicability, it was tested on a comprehensive data set collected from 10 commercial dairy farms. The added value of the developed method can be summarized as the ability to improve the quality of huge data sets with complex structure by distinguishing implausible observations from observations indicating physiological extreme conditions. The underlying concept can be applied to future data collections in science as well as in agricultural practice with regard to precision livestock farming.

**Abstract:**

The aim of this work was to develop an innovative multivariate plausibility assessment (MPA) algorithm in order to differentiate between ‘physiologically normal’, ‘physiologically extreme’ and ‘implausible’ observations in simultaneously recorded data. The underlying concept is based on the fact that different measurable parameters are often physiologically linked. If physiologically extreme observations occur due to disease, incident or hormonal cycles, usually more than one measurable trait is affected. In contrast, extreme values of a single trait are most likely implausible if all other traits show values in a normal range. For demonstration purposes, the MPA was applied on a time series data set which was collected on 100 cows in 10 commercial dairy farms. Continuous measurements comprised climate data, intra-reticular pH and temperature, jaw movement and locomotion behavior. Non-continuous measurements included milk yield, milk components, milk mid-infrared spectra and blood parameters. After the application of the MPA, in particular the pH data showed the most implausible observations with approximately 5% of the measured values. The other traits showed implausible values up to 2.5%. The MPA showed the ability to improve the data quality for downstream analyses by detecting implausible observations and to discover physiologically extreme conditions even within complex data structures. At this stage, the MPA is not a fully developed and validated management tool, but rather corresponds to a basic concept for future works, which can be extended and modified as required.

## 1. Introduction

In the era of ‘Big Data’ it is possible to record large amounts of data automatically and cost-effectively. Irrespective of the field of research, data quality plays a major role in statistical analysis. As will be shown by means of several examples, the acquisition of automated mass data in animal sciences carries the risk that technically induced disturbances lead to values that leave the physiological range and are therefore implausible. Nevertheless, it is possible that physiologically extreme values occur, for example, when evoked by a certain treatment in the course of an experiment, because of diseases, or natural extreme situations such as in the peripartum period.

As an example of recorded data exceeding the physiologically normal range and becoming extreme, clinical mastitis of lactating cows can be mentioned. Fogsgaard et al. [1] demonstrated that the body temperature increased massively about 18 h after an experimentally induced infection with *Escherichia coli* and rectal temperatures of about 40 °C were reached. At the same time, the authors observed an extreme increase in the somatic cell count (SCC) of the milk, as well as a decrease in feed intake and milk performance. In the case of clinical ketosis, measurable physiological changes can occur in early lactation. Known characteristics are a decrease in feed intake and milk performance as well as an increase in ketone bodies in the blood, in particular non-esterified fatty acids (NEFA) and β-hydroxybutyrate (BHB) [2]. Claw disorders can also be mentioned as a further example. Pavlenko et al. [3] observed significantly lower milk performances and a different standing and lying behavior in cows with digital dermatitis compared to healthy animals. In this context, further diseases could also be mentioned, such as milk fever and a displaced abomasum.

From these examples, it becomes clear that disease-related extreme values can occur simultaneously or with a time delay between observations in various measurable parameters. Such observations can generally be classified as plausible and should not be classified as outliers or erroneous observations from a physiological point of view. For mastitis, ketosis and claw diseases, for example, estimated incidences of 21.6%, 1.7% and 19.5% of affected lactations can be found [4]. Therefore, it is likely that physiologically extreme values caused by such diseases will occur in any data collection. Additionally, other factors may induce real extreme values, such as management incidents, stress, unwanted restriction access to water and feed, extreme climatic conditions or heat as they may affect behavior, milk production or intake and consequently other measurable traits.

Real extreme observations or recording errors can be found in both test day milk yields and milk components. In this context, Wiggans et al. [5] and Gao et al. [6] have established strategies for handling such extreme observations. Wiggans et al. [5] developed a procedure in which preceding and subsequent observations of test day records were investigated with a univariate analysis to detect implausible observations. In comparison, Gao et al. [6] used a multivariate method based on the Mahalanobis distance between fixed effect corrected test day milk performances and protein and fat contents.

In addition to true extreme values, there is the risk of technical random or systematic errors, in every automated sensor-based data acquisition, e.g., with transponder-assisted feed and water intake, sensor-based rumination and locomotion activity measurements or the intra-reticular recording of pH and temperature. In the case of feed intake measurements in pigs, Eissen et al. [7] presented algorithms based on threshold-based rules to detect implausible observations. The random non-directional drift observed during in continuous intra-reticular pH measurements was discussed by Villot et al. [8]. The authors solved this problem of systematic errors by applying a trend correction with moving averages to calculate relative moving mean centered pH values which served as indicator for subacute ruminal acidosis (SARA). However, there are also approaches to further discriminate the source of extreme observations. Alawneh et al. [9] for example investigated the daily walkover live weight of cows and differentiated identified outliers into ‘biologically implausible’ or ‘potentially erroneous’. The authors made these classifications based on a smoothed live weight curve, where observations outside the ±4 standard deviation (SD) interval were classified as ‘biologically implausible’ and observations outside the ±1.96 SD interval (95% confidence interval) as ‘potentially erroneous’ cases.

Since the measured values in the statistical evaluations are assumed to be random variables, outlier detections based on the mean ±3 empiric estimated standard deviations ($\bar{x}\pm 3\hat{\sigma }$) are often found, as in the study by Liang et al. [10], where daily reticular temperatures were investigated. Another example is mid-infrared (MIR) spectral data, which contains 1060 variables per sample when measured with a Foss spectrometer (Foss, Hillerod, Denmark). With such data, it is common to use the standardized Mahalanobis distance (global H-value, GH), whereby spectra with a GH > 3 are regarded as outliers [11,12]. Another common method is to detect outliers within the framework of statistical modeling using standardized or studentized residuals between measured and predicted values [13]. As a rule of thumb, the observations with standardized or studentized residuals of ±3 are regarded as outliers. Regarding the threshold, however, different recommendations can be found in the literature. Rousseeuw et al. [14], for example, proposed 2.5 as the threshold for outlier classification.

Therefore, without a global visualization of the data it is difficult for specialists to discriminate extreme records and erroneous data. This issue is even more highlighted when data are analyzed by general statisticians having no skills in animal sciences.

The main objective of this study was to develop an innovative approach of data cleaning based on a multivariate plausibility assessment (MPA) of simultaneously recorded data, considering both automatically collected sensor data and conventional samples such as milk and blood samples. The aim was to distinguish in an automated way between ‘physiologically normal’, ‘physiologically extreme’ and ‘implausible’ observations. The developed MPA procedure is applied on data collected in agricultural practice in order to obtain an overview of the data quality of the individual measuring systems and to prepare the dataset for downstream analyses. At this stage, the MPA is not a fully developed and validated management tool. It rather corresponds to a basic concept which has been adapted to the available data set, but which can be extended and modified as required in future works.

## 2. Materials and Methods

### 2.1. Ethical Declaration

The experiment was conducted in accordance with the German legislation on animal protection (Animal Welfare Act) and was approved by the Lower Saxony State Office for Consumer Protection and Food Safety (LAVES, Oldenburg, Germany; AZ: 33.9-42502-05-17A106).

### 2.2. Data Recording

The data collection was carried out as part of the project ‘Evaluation of Animal Welfare in Dairy Farming—Indicators for the Metabolism and Feeding’ (IndiKuh). The project focused on metabolic and digestive disorders of early lactating cows and aimed both to validate existing and to develop novel indicators. In this regard, the investigations targeted the disorders sub-clinical ketosis and subacute ruminal acidosis, which is why a wide range of different data was collected.

#### 2.2.1. Farms and Animals

**Figure 1:**
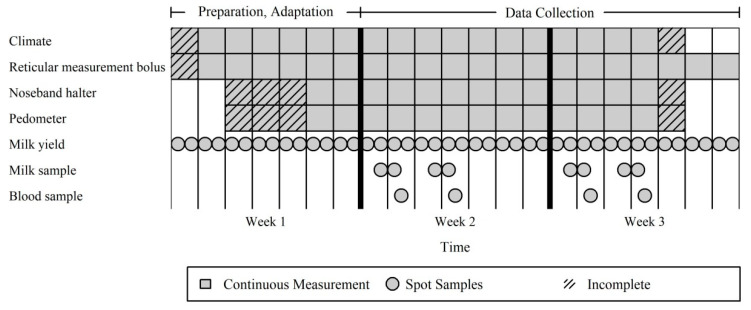
Data collection scheme used in each of the 10 farms.

The data collection was carried out in 10 commercial dairy farms in the northwest of Lower Saxony, Germany, between April 2017 and March 2018. The farms are characterized by 305 days (d) milk yields between 9200 and 11,100 kg and approximately 200 to 600 cows, and therefore can be classified as above average with regard to performance and size in Germany. Each farm was visited for 3 weeks, whereof the first week was used for preparations and the animals’ adaptation to the measuring instruments. In the following two weeks, the data recording took place. The data collection scheme used in each farm is illustrated in Figure 1. On each farm, 10 Holstein cows in early lactation were selected. Attempts were made to ensure that all parities of the cows (1, 2, 3 and ≥4) were covered equally in the farms. All farms had a loose-housing outdoor climate stable with resting pens and fed a partially or totally mixed ration with no change in diet during the 3 weeks stay. In each farm the cows were milked twice a day.

#### 2.2.2. Climate Data

On each farm, the temperature and relative humidity in the stable were recorded in 15 min intervals using three to six Tinytag climate data loggers (Tinytag Plus 2 TGP-4500, Gemini Data Loggers, Chichester, UK). The data loggers were positioned at a height of about 2 m in the feeding, lying and drinking areas. In addition, climate data were obtained from the German Weather Service [15], which was collected at the weather station in Altenoythe, Germany, in the vicinity of the 10 farms.

#### 2.2.3. Intra-Reticular pH and Temperature

In total, 100 cows were equipped with an eCow measurement bolus (eCow Ltd., Exeter, Devon, UK) for the intra-reticular continuous measurement of the pH and the temperature. Before insertion, all boluses were activated in a water bath at 38.4 °C and calibrated in pH 4 and pH 7 buffer solutions. The raw pH and temperature data were provided as mean values of 15 min time periods by the eCow boluses (termed ‘boluses’ in the remainder of this paper).

#### 2.2.4. Eating, Chewing and Locomotion Behavior

The jaw movement behavior was recorded using noseband-sensor halters (RumiWatch, ITIN + HOCH GmbH, Liestal, Switzerland). Simultaneously, pedometers (RumiWatch) were equipped to measure locomotion behavior. They were attached to the rear left or right leg. The data recording started at the end of the first week and continued during the following two weeks. The raw data of the RumiWatch noseband sensor and the pedometers were converted using the RumiWatch Converter V0.7.4.13 (RumiWatch) into a 1 h resolution.

#### 2.2.5. Milk Yield and Milk Samples

The milk yield of all animals was measured with the on-farm milk quantity recording system. In addition, four evening milk samples and four morning milk samples of the following day were taken during the data collection period. Samples were collected following ICAR guidelines [16] and were preserved with Bronopol (Georg Hansen e.K., Wrestedt, Germany). The samples were analyzed by the Landeskontrollverband Weser-Ems e.V. in Leer, Germany. In addition to the standard components such as fat, protein, lactose, urea and somatic cell count (SCC), the milk MIR spectra were also provided. The somatic cell count was measured using a flow cytometer (Fossomatic FC, Foss). The raw spectral data, which were recorded with a spectrometer (MilkoScan FT+, Foss) were then standardized according to Grelet et al. [17] using the tools and strategies developed at the Walloon Agricultural Research Center (CRA-W, Gembloux, Belgium).

#### 2.2.6. Blood Samples

Within the data collection period, blood samples from each cow were taken 4 times from the vena caudalis mediana after the a.m. milking. Samples were collected in separate tubes with serum clot activator, one for BHB and NEFA and the other for glucose (GLU) analysis, and were centrifuged to harvest serum. The analysis was conducted in the laboratory of the Institute of Veterinary Medicine at the University of Goettingen.

Since the data collection was carried out under field conditions, some individual recordings were disrupted unintentionally for some of the collection systems. These include, for example, unreadable boluses, noseband halters and pedometers.

### 2.3. Data Preparation

#### 2.3.1. Unification of the Temporal Resolution

**Figure A1:**
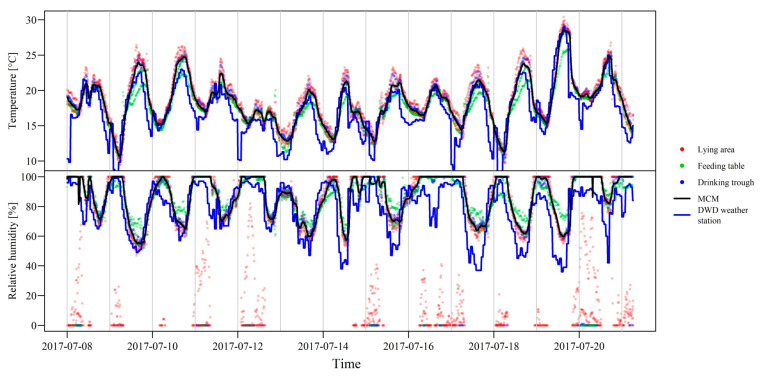
Climate data of a farm. Plotted are the individual observations of the Tinytag climate loggers from different positions in the stable as well as the hourly temperature data of a nearby weather station of the German Weather Service [15]. The black line corresponds to the moving convolutional median (MCM) of the individual Tinytag climate loggers.

Climate data recorded simultaneously with three to six Tinytag loggers at different locations in the stable displayed very similar values. Nevertheless, some loggers showed outliers particularly for the relative humidity, e.g., single observations with a relative humidity of 0%. Since both the determination of mean- or median-based daily courses led to distorted results, a moving window-based method was used taking into account all simultaneous Tinytag measurements in order to get the best estimate of the temperature in the stable. Thereby, all Tinytag time series were summarized in a moving convolutional median with a window length of nine values, 2 h respectively, to obtain one averaged course for temperature and one for relative humidity. Since the time window is relatively small in relation to the rate of change of the measurements, no significant smoothing behavior was observed with this method, so that the variance of the values was only marginally influenced (see Figure A1 for an example in the Appendix A).

The multivariate plausibility check of the aforementioned measured variables is challenging, as some of them differ in their temporal resolutions. Blood samples with daily values have the coarsest resolution in data collection. For the multivariate plausibility check, all continuous measurements were therefore also reduced to values on a daily basis.

Theoretically, the bolus data should consist of 96 observations per day, and the halter and pedometer data of 24 observations per day. In practice this was not always the case. Among the 96 readable boluses, two devices recorded on average only 41 and 66 values per day throughout the entire data collection period due technical failure. The same was observed for the halter and pedometers, where, for example, cases were detected with 15 of 24 observations per day. This led to difficulties in the aggregation of these time series to values on a daily basis, where mean-based values should be determined for the bolus measurements (pH or temperature daily mean or median) and the sum of events over the day for the halter and pedometer data (daily duration of rumination, feeding and lying). Since missing observations have less influence on averaging than summation, up to 2 h missing observations were allowed for the bolus data 24 h averaging, and only 1 h for the halter and pedometer data 24 h summation. If not enough data per day were available, the data of the affected traits from this day were set to missing and thus excluded from further analysis.

The milk MIR spectra consisting of transmittance values (T) were converted into absorbance values (A) using a logarithm function with A = −log10(T) as common in spectroscopy [18]. In the remainder of this paper, only absorbance spectra were considered. To aggregate the evening and the subsequent morning milk samples, the components as well as the MIR spectra were daily averaged with a weighted average proportionally to the milk yield. The aggregated values were then assigned to the day of the evening milking. The blood values were also assigned to the previous day to compensate for the latency of the animal’s digestion and metabolism.

After all the preparation steps mentioned above, the data were merged into one data set with a daily resolution. Since all continuous measurement systems were installed from day 5 at the latest, the data from day 6 to day 21 were considered for the plausibility assessment, so that 16 observations per animal were available on a daily basis.

#### 2.3.2. Handling Missing Values

As the daily average temperature in the stable is going to be used in the MPA and some observations are missing, the extremely high correlation between the external climate data from the weather station and the stable climate data were used to predict missing values. For this a linear model (R^2^ = 0.99, residual standard error (RSE) = 0.67 °C) was used, in which the temperature in the stable was modeled as a function of the outside temperature and a farm effect.

In around 6% of the cases of days where milk samples were taken, the separation of the total daily milk yield into morning and evening yield was missing. However, this information was needed, for example, to determine the pooled MIR spectra. As this only concerned a few individual cases per animal, all available observations were used to estimate the morning and evening milk quantity for affected days using the available daily total milk yield. This took into account that the morning milk yield corresponds to about 55% of the daily total milk yield when milking twice a day [19]. Thus, a linear model (R^2^ = 0.94, RSE = 1.34 kg/d) was used to estimate the morning milk performance considering an animal effect as well as the interaction between animal effect and total milk performance. The evening milk yield was then estimated by subtracting the estimated morning milk yield from the daily milk yield.

#### 2.3.3. Transformations

Since both the BHB and NEFA values in the blood as well as the SCC are extremely right-skewed, the former were Log10 transformed and the latter were transformed by calculation of the somatic cell score (SCS) according to Wiggans and Shook [20] using the following formula:

(1) $$SCS={log}_{2}\left(\frac{SCC}{100}\right)+3$$

These transformations resulted in an approximately normal distribution of all three variables.

In 3 of the 100 cows, medications affecting rumen fermentation were administered by herd managers due to unspecified clinical signs in or immediately prior to the experimental period. All animal-individual observations of these cows after administration of the medications were discarded to the end of data collection.

### 2.4. Multivariate Plausibility Assessment

#### 2.4.1. Concept and Underlying Assumptions

The developed MPA concept for checking of simultaneously measured data is based on the fact that when real extreme values occur, they are reflected not only in one but most likely in several measurable traits. Therefore, the following assumptions were made:

- Several of the measured traits are (physiologically) associated
- Cases of disease are reflected in more than one of the measured parameters in form of conspicuously high or low values
- The probability that two or more measuring systems will display erroneous values at the same time is close to zero

**Figure 2:**
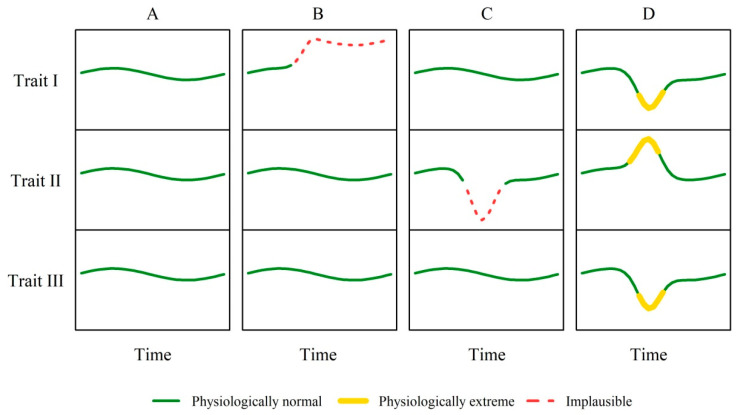
Simulated examples illustrating the classifications ‘physiologically normal’, ‘physiologically extreme’ and ‘implausible’ for observations in three physiologically associated traits.

Consequently, it can be concluded that if extreme observations are observed in only one trait, they are most likely not of a physiological nature but rather due to an error in data recording. Furthermore, in the absence of extreme observations, all are considered to be plausible. Therefore, three possible classifications are assumed whereby ‘physiologically normal’, ‘physiologically extreme’, and ‘implausible’ cases are distinguished. This classification is illustrated in Figure 2 by 4 simulated case studies taking into account 3 traits in the form of time series data:

- Case A represents a healthy and thus physiologically normal animal,
- Case B is a cow, whose reticular pH sensor had a malfunction with pH as Trait I,
- Case C is a cow with a temporarily dropped halter with daily rumination duration as Trait II, and
- Case D shows a cow with a clinical mastitis, where Trait I would be the milk performance, Trait II the SCC or body temperature and Trait III the daily eating or rumination duration.

In summary, simultaneously collected characteristics are used for mutual plausibility checks in order to ensure adequate data quality for downstream analyses.

#### 2.4.2. Selection of Traits

Since a large number of traits could be derived from the collected data, a target-oriented selection had to be made, because a drift occurring in one sensor is likely to affect all the data collected by it. For all variables measured with a common measuring system, traits were selected that were as independent and uncorrelated as possible. For the bolus data, the daily average pH ($r.\bar{pH}$) and the median of the reticular temperature ($r.T_{med}$) were selected, since the pH and temperature measurements were recorded with two different sensor systems in the bolus. For the temperature, the median was taken as a robust statistic for the daily average instead of the arithmetic mean, since the temperature measurement in the reticulum is temporarily strongly influenced by the water intake [10]. The use of the arithmetic mean, however, would lead to an underestimation of the daily average temperature. Regarding the halter data, the total eating and rumination duration (Σ.Rt and Σ.Et) were chosen. For the pedometer data, only the total lying duration (Σ.Lt) was selected, since, for example, lying and standing time are highly correlated (r = −0.99). With regard to performance and milk parameters, the daily milk yield (MY), the SCS and the entire MIR spectrum was used. As already stated by Gengler et al. [18], the special feature of the MIR spectrum is that it can be seen as a ‘fingerprint’ of the entire milk composition and thus contains not only information on the standard components (fat, protein, lactose, urea) but also on fine milk composition such as the fatty acid pattern of the milk fat. For this reason, solely the MIR spectrum of milk will be considered in the following. Furthermore, all three blood parameters were taken into account, because the analyses for BHB and NEFA were performed on the same sample and are only slightly correlated (r = 0.13), and because glucose was determined independently on a separate blood sample.

#### 2.4.3. Technical Implementation of the MPA

Due to the experimental design, the available data are subject to a hierarchical structure with the levels ‘farm’, ‘animal’ within the respective farm and the ‘repeated measurements’ within each animal. In addition, there are other influencing factors, such as the lactation stage and parity, which can also have an effect on the variables under investigation. Since the data collection covered a period of one year, the climate could also have an effect on the respective parameters. Heat stress can lead to a decline in the rumination duration, feed intake and milk performance [21]. For this reason, the temperature in the stable ($s.\bar{T}$) was also taken into account as a possible abiotic influencing factor. As the MPA approach is based on deviations of observations from expected values both on animal and single measurement level, these deviations have to be quantified first. To consider both the hierarchical data structure and the fixed effects, linear mixed models were established as follows for all traits except for the milk MIR spectrum:

(2) $$y_{ijklm}=\beta_{0}+\beta_{1}s.{\bar{T}}_{ijklm}+P_{i}+\sum_{p=1}^{4}\left(\beta_{p}{DIM}_{ijklm}\times P_{i}\right)+F_{j}+F_{j}\times {TD}_{k}+A_{l}+e_{ijklm}$$

where $y_{ijkl}$ is the observation m of cow l on the farm j for trait y, $s.{\bar{T}}_{ijklm}$ is the daily mean temperature in the stable, $P_{i}$ is the fixed effect of parity class i (1, 2, 3 or ≥4), and ${DIM}_{ijklm}\times P_{i}$ is the interaction of lactation stage and parity. $\beta_{0}$ is the intercept, while $\beta_{1}$ and $\beta_{p}$ are regression coefficients. The farm $F_{j}$, the interaction of farm and the test day $F_{j}\times {TD}_{k}$ and the animal $A_{l}$ are considered as normally distributed random effects with $F_{j}\sim N\left(0,\sigma_{F}\right)$, $F_{j}\times {TD}_{k}\sim N\left(0,\sigma_{F\times TD}\right)$ and $A_{l}\sim N\left(0,\sigma_{l}\right)$. Furthermore, $e_{ijklm}$ is a random error with $e_{ijklm}\sim N\left(0,\sigma_{e}\right)$.

The cases shown in Figure 2 are limited to the hierarchy level of repeated measurements within an animal. However, considering the data structure and the modeling, four different scenarios are conceivable, especially for the bolus-, noseband halter- and pedometer-derived traits:

- Neither the animal effect nor the residuals within the animal are extreme,
- The animal effects are extreme, but not the residuals within the animal,
- The residuals within one animal are extreme, but not the animal effect,
- Both the animal effect and the residuals within the animal are extreme.

The modeling assumes that both the animal effects and the residuals are normally distributed and with a mean of zero. With regard to the classification of the aforementioned four cases, two new binary coded auxiliary variables $A^{\prime }$ and $e^{\prime }$ were added for all traits based on the level of the animal effects as well as on the level of the residuals. Animal effects and residuals were classified as extreme and coded as 1, if the estimated effects were outside the confidence interval of ±3 $\hat{\sigma }$ with $\hat{\sigma }$ being the empirically estimated standard deviation of the residuals or animal effects; otherwise they were classified as normal and coded as 0. If ≥50% of an animal’s residuals were extreme, the animal effect was also coded 1 in the auxiliary variable. However, the coding for the animal effect $A^{\prime }$ was only done for the bolus-, noseband halter- and pedometer-derived traits, since in these data collections the measuring instruments were confounded with the respective animal. The 3 $\hat{\sigma }$ wide confidence range was chosen, as it is a generally accepted threshold [13]. According to the 3 σ rule, one would therefore expect 0.3% of the data as outliers for the random animal effects as well as for the residuals with true normal distributions.

A special case is the consideration of the MIR spectral data in this plausibility assessment. Of the 1060 absorbance values of the different wavelengths, the noisy areas were removed first according to Grelet et al. [17]. To detect extreme spectra, a principal component analysis (PCA) based on the reduced spectra was performed as described by Soyeurt et al. [22] to determine the number of components that explain 99% of the variability. The GH was then determined from the scores of the required principal components (n = 7). Spectra with GH > 3 [11,12] were coded as 1 and otherwise 0 in a further auxiliary variable GH’. If more than 50% of the spectra of an animal had a GH > 3, an additional animal-based auxiliary variable for the GH would also have to be coded as 1. However, such a case did not occur.

**Figure 3:**
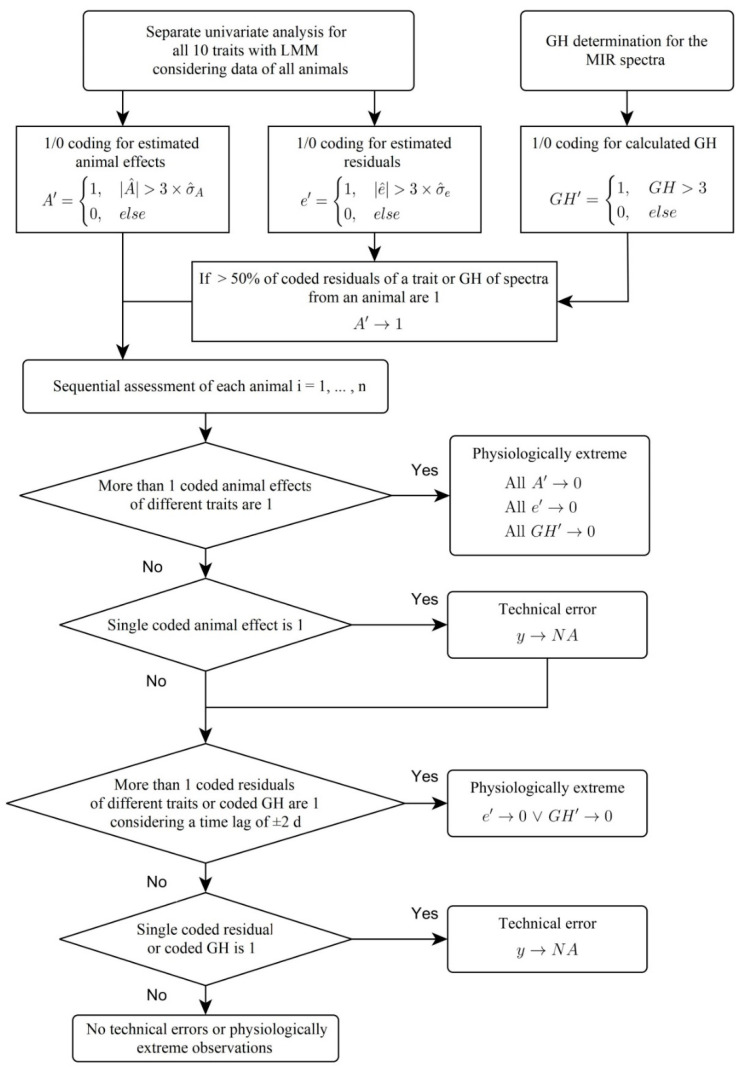
Flow chart demonstrating the pseudo-code of the developed multivariate plausibility assessment. GH = global H, standardized Mahalanobis distance, MIR = mid-infrared, NA = not available, y = trait, $\hat{\sigma }$ = empirically estimated standard deviation of the residuals or animal effects.

After 1/0 coding for all 11 traits on the individual observation and entire animal level, the procedure described in the section “Concept for Multivariate Plausibility Assessment” had to be applied. The developed R [23] program is visualized in Figure 3 in form of pseudo code in a program flow chart. This figure was created using the yEd graph editor [24]. Time lags ± 2 d were also taken into account in the MPA, since delayed extreme value formation can occur (e.g., [1]). This means that extreme observations of at least two different traits were classified as physiologically extreme if they were not only observed on the same day but also in a time window of ±2 d.

Unless otherwise stated, all data preparation, creation of figures and statistical analysis was done within the software environment R [23]. The package lme4 [25] was used for regression parameter estimation of linear mixed models.

## 3. Results and Discussion

### 3.1. Classification of Observations and Descriptive Statistics

**Figure 4:**
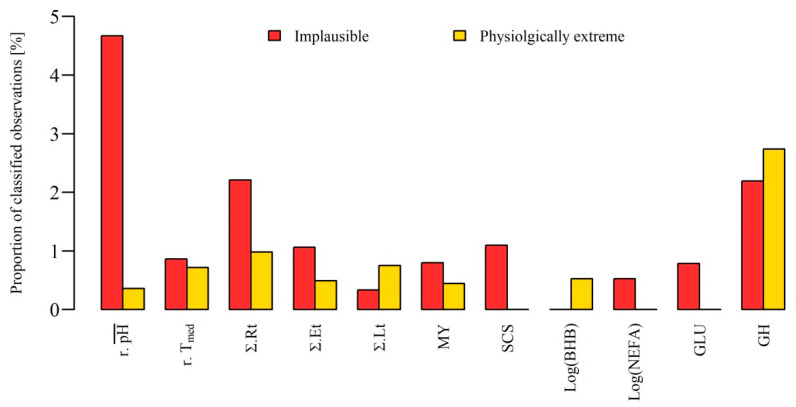
Proportion of classified observations assigned by the multivariate plausibility assessment for the classes ‘physiologically extreme’ and ‘implausible’ of all available observations for all 11 evaluated traits. BHB = β-hydroxybutyrate in blood, GLU = glucose in blood, GH = global H, standardized Mahalanobis distance of milk mid-infrared spectra, MY = daily milk yield, NEFA = non-esterified fatty acids in blood, $r.\bar{pH}$ = ruminal daily mean pH, $r.T_{med}$ = median of the reticular temperature, SCS = somatic cell score according to Wiggans and Shook [20], Σ.Et, Σ.Rt and Σ.Lt = daily duration of eating, rumination and lying.

To assess the performance of the developed concept of a MPA for automated data acquisition systems, it was applied on data collected under field conditions. Thereby, it was examined which proportions of the data considered in the analysis were classified as ‘physiologically normal’, ‘implausible’ or ‘physiologically extreme’ observations. The result of this classification is illustrated in Figure 4 by showing the relative frequency of ‘implausible’ and ‘physiologically extreme’ observations for each trait. In addition, Table 1 provides an overview of the descriptive statistics of the data set before and after the MPA. Observations of traits classified as ‘implausible’ were set to missing in the data set.

For almost all traits, both classifications were made for the data set. In particular, the pH values derived from the data recordings with boluses showed the highest relative frequency of implausible observations with almost 5%. For all other traits less than 2.5% observations were classified as implausible. Comparing the relative frequency of classified ‘physiologically extreme’ and ‘implausible’ milk performances with 1.2% of all observations to the findings of Wiggans et al. [5], who identified 1.9% abnormal test day milk records, the same order of magnitude for conspicuous observations can be identified. In other automatic measurements, such as walkover live weight recording, Alawneh et al. [9] found a much higher frequency of conspicuous observations. The authors detected a total of 12% outliers, of which they classified 25% as ‘biologically implausible’ and 75% as ‘potentially erroneous’. In comparison, Eissen et al. [7] found incorrect observations in about 6% of the automatically recorded feed intakes of pigs. Consequently, the MPA algorithm presented here classified similar proportions of conspicuous observations with regard to the various traits on the basis of the example data set.

Before applying the MPA, 4 of the 100 boluses were not readable at the end of the trial, and thus the data could not be downloaded. Two further erroneous boluses were also removed before the MPA as they had on average only 41 or 66 observations per day instead of the 96 theoretical observations. In addition, the observations from another cow were removed, as fermentation-influencing medication was administered immediately before data collection. The MPA, however, classified the data of four other boluses as implausible, so that only reticular pH data of 89 of the 100 animals were available for further analysis. In the data set examined here, it is remarkable that especially the reticular pH measurements already had the biggest losses during data collection and showed the most implausible values in the MPA. At this point, it has to be emphasized that intra-reticular measurement is one of the most complicated and innovative measurement methods used in this study. This is partly due to the milieu in which the bolus is located, the generally sensitive pH measurement electrodes and the fact that each bolus is confounded with the animal and cannot be calibrated after data recording.

In some studies, spectra with a GH > 3 are considered to be outliers and are excluded from further analyses because in the development of MIR-based prediction equations they could endanger the robustness and accuracy [11,12]. However, in this study, 10 of the 18 MIR spectra with a GH > 3 were classified as ‘physiologically extreme’, and thus were accompanied by extreme observations in other traits in the present study. It shows that removing records based only on spectral distances can erroneously deprive the dataset from real extreme values that potentially could be interesting for model development. This also confirms the fact that the fine milk composition reflects the physiological status of the cow, as demonstrated by Grelet et al. [26] and Colman et al. [27] for the metabolic status or digestive disorders like SARA.

In total, about 1% of the data could be rescued that would have been classified and removed as outliers in common univariate plausibility checks, but which could be assigned to physiological extreme conditions by the developed MPA.

### 3.2. Case Studies

**Figure 5:**
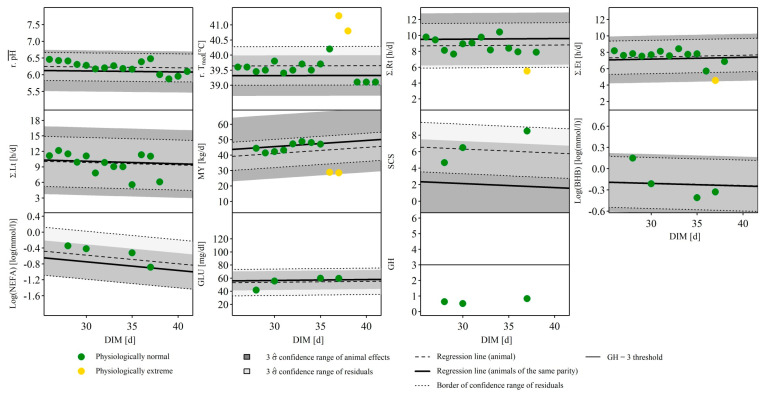
Case study of a cow of 3rd parity with signs of a clinical mastitis. The observations of 11 different traits as a function of the lactation stage, investigated in the multivariate plausibility assessment, are illustrated. BHB = β-hydroxybutyrate in blood, DIM = days in milk, GLU = glucose in blood, GH = global H, standardized Mahalanobis distance of milk mid-infrared spectra, MY = daily milk yield, NEFA = non-esterified fatty acids in blood, $r.\bar{pH}$ = ruminal daily mean pH, $r.T_{med}$ = median of the reticular temperature, SCS = somatic cell score according to Wiggans and Shook [20], Σ.Et, Σ.Rt and Σ.Lt = daily duration of eating, rumination and lying, $\hat{\sigma }$ = empirically estimated standard deviation of the residuals or animal effects.

To illustrate the applicability of the MPA, Figure 5 visualizes the collected data in the form of time series for a cow of the 3rd parity showing signs of clinical mastitis in several of the traits investigated. Plotted are all 10 variables as well as the GH of the spectra as a function of time. In addition to the individual observations on a daily basis, the estimated regression line of the respective trait was plotted as a function of DIM for all animals of the same parity. The estimated random effect of the farm was taken into account to determine the intercept of the regression line. For illustrative purposes, the temperature in the stable which was also considered as a covariable in the model was mean centered and set to 0 for the prediction of the line. Around this regression line, the 3 $\hat{\sigma }$ confidence range of estimated random animal effects was drawn in dark grey. The regression line of the animal, for which both the estimated random farm effect and the random animal effect were considered for the determination of the intercept, was added as a black dashed line. This individual animal line is also surrounded by the 3 $\hat{\sigma }$ confidence range of the residuals in light grey, which is enclosed by dashed black lines. This figure shows that from the 35th DIM onwards, the reticular temperature and the somatic cell counts in the milk temporarily increase, and that the milk performance, the rumination and eating duration decrease. Except for the SCS, all measured variables simultaneously show extreme values and are classified as ‘biologically extreme’ by the MPA. It should also be noted that SCS, which is generally considered to be an essential indicator of mastitis, is not classified as ‘biologically extreme’ for these observations due to the generally high somatic cell counts of this animal and the overall high residual variance of all animals for this trait. If the dataset had been univariately plausibilized using standardized or studentized residuals, the ‘biologically extreme’ observations of the other four traits would have been removed from the dataset. However, since the MPA considered all variables in a multivariable procedure, the observed extreme values could be classified as plausible. The observations made here thus correspond to known signs of clinical mastitis and are in line with the results of Fogsgaard et al. [1], who induced mastitis in cows with *Escherichia coli* experimentally.

**Figure 6:**
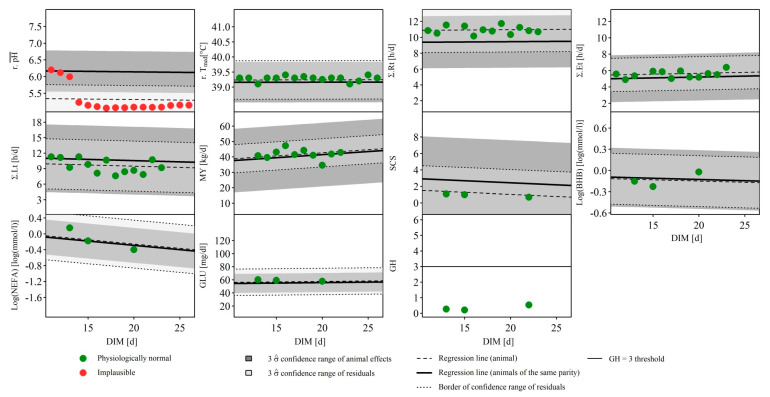
Case study of a cow of 3rd parity with erroneous intra-reticular pH measurement. The observations of 11 different traits as a function of the lactation stage, investigated in the multivariate plausibility assessment, are illustrated. BHB = β-hydroxybutyrate in blood, DIM = days in milk, GLU = glucose in blood, GH = global H, standardized Mahalanobis distance of milk mid-infrared spectra, MY = daily milk yield, NEFA = non-esterified fatty acids in blood, $r.\bar{pH}$ = ruminal daily mean pH, $r.T_{med}$ = median of the reticular temperature, SCS = somatic cell score according to Wiggans and Shook [20], Σ.Et, Σ.Rt and Σ.Lt = daily duration of eating, rumination and lying, $\hat{\sigma }$ = empirically estimated standard deviation of the residuals or animal effects.

**Figure A2:**
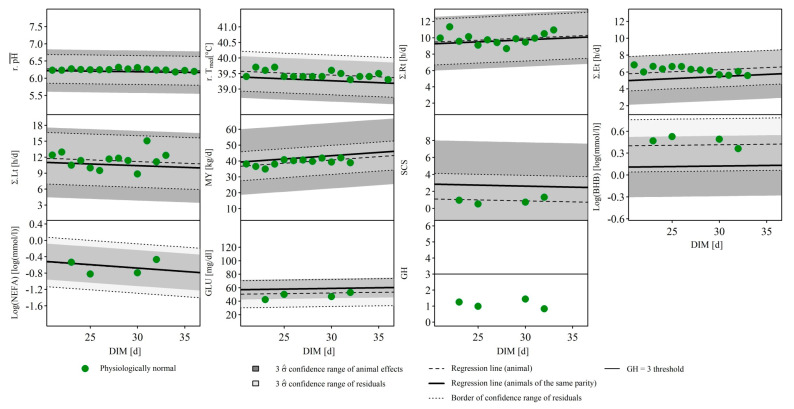
Case study of a cow of 5th parity in which all observations were classified as ‘physiologically normal’. The observations of 11 different traits as a function of the lactation stage, investigated in the multivariate plausibility assessment, are illustrated. BHB = β-hydroxybutyrate in blood, DIM = days in milk, GLU = glucose in blood, GH = global H, standardized Mahalanobis distance of milk mid-infrared spectra, MY = daily milk yield, NEFA = non-esterified fatty acids in blood, $r.\bar{pH}$ = reticular daily mean pH, $r.T_{med}$ = median of the reticular temperature, SCS = somatic cell score according to Wiggans and Shook [20], Σ.Et, Σ.Rt and Σ.Lt = daily eating, rumination and laying time, $\hat{\sigma }$ = empirically estimated standard deviation of the residuals or animal effects.

As a further example, Figure 6 visualizes the data of a cow of the third parity in which the intra-reticular pH measurement clearly failed. All other variables show observations classified as ‘physiologically normal’. A further example with only observations classified as ‘physiologically normal’ can be found in Figure A2 in the Appendix A.

### 3.3. Further Remarks and Implications for Future Research

On the basis of the data considered here and most of the studies mentioned in the introduction, it can be concluded that automated data acquisition often produces conspicuous observations which are either physiologically justified or caused by technical errors. In the age of ‘Big Data’, the possibility of direct data control is not feasible due to the volume of data, so that the need for systematic and, above all, automatable monitoring strategies to ensure data quality is increasing. Such data should therefore be examined in detail before being further analyzed in the context of a specific objective.

With an extensive data set from agricultural practice, there is a risk that extreme values due to different physiological causes may occur. Considering the estimated incidences of diseases such as mastitis, claw diseases or ketosis (see [4]), the probability of finding such cases in a data set such as the present one is therefore relatively high. Looking at the case shown in Figure 5, for example, it can be seen that clinical mastitis is accompanied by a temporary decrease in the duration of rumination and eating. It is highly probable that this will be accompanied by a decrease in dry matter intake. This would have the consequence that the rumen fill level decreases and also the fermentation process, and thus the concentration of short-chain fatty acids decreases [28]. This would explain the temporary increase of the reticular pH value, which is an associated trait of the ventral ruminal pH value. Therefore, those values physiologically make sense and may be considered in further analysis, whereas a univariate data cleaning process would have removed these records. Additionally, for further analysis with a specific objective it allows a valuable deep understanding of the data and of such complex and interdependent mechanisms occurring, especially if the data were collected in the field and thus not under experimental standardized conditions.

One of the biggest advantages of the MPA presented here is that the classification depends on the distributions of the investigated variables, whereby the entire hierarchical data structure could be considered. Consequently, no static thresholds had to be chosen subjectively, as in the case of Denwood et al. [29], who, for example, removed reticular measured pH values ≥ 10 before analysis.

Interestingly, the concept presented here is also similar to the Heatime^®^ System (SCR Ltd., Netanja, Israel), in which the simultaneous measurement of neck activity and chewing behavior is used for heat detection. Further resemblances exist to the method of Van Hertem et al. [30], who developed statistical models for the detection of lameness using multivariate sensor data such as milk performance, rumination behavior, and neck activity. Furthermore, the quality control charts described for the first time by Shewhart [31] are based on a similar concept. These charts are frequently used in industry for quality management in the monitoring of processes and provide a tool to check whether a statistical measure is under control or not. For this purpose, distribution-dependent thresholds are also used like $\bar{x}$ ± 3 SD.

The remaining question is how to deal with the result of the classification of observations into ‘physiologically normal’, ‘implausible’ or ‘physiologically extreme’. This depends on the research question. The MPA presented here offers the possibility to automatically locate conspicuous observations. Those classified as biologically extreme should be investigated in particular, as extreme observations are often of scientific interest. However, the observations classified as ‘implausible’ are likely to occur due to technical and systemic errors in data collection and should be discarded in further analysis. The added value of the method presented here is that on the one hand the data quality can be improved and on the other hand physiologically interesting cases can be detected automatically. One condition for success, however, is that a sufficient number of traits are collected to cover the majority of disease-related changes.

In future studies, other traits are conceivable to be taken into account for such a plausibility assessment of simultaneously collected data. For instance the electrical conductivity of milk as an auxiliary characteristic for udder health (e.g., [32]) or the development of body weight as it is related to the energy balance (e.g., [9]). In this respect, data collection in the dairy sector using automatic milking systems in combination with other monitoring systems, such as for heat detection, is of particular interest. As already summarized by Jacobs and Siegford [33], the use of such a technology facilitates recording of numerous traits by means of different sensor systems, which, if properly taken into account, could lead to many benefits for the economy and animal welfare in dairy farming. It is possible that our MPA procedure can be applied to such data collections to ensure data quality and to detect physiologically extreme conditions.

### 3.4. Limitations of the Study

The presented MPA should not be seen as a fully developed management tool. At the current stage, the system should be considered more as a warning system to highlight potentially implausible data or observations related to extreme physiological conditions than as a validated cleaning system for routine data. What is decisive is the underlying concept, in which data collected with different sensor systems can be used for mutual plausibility checks. The modeling used in this study was customized to the data set that was used as an example. It is possible to adapt the modeling individually for even more extensive data sets. On the one hand, this could be relevant if not only data from early lactation but from the entire lactation are available. Then, typical lactation curves, such as for milk yield, milk ingredients, energy balance or dry matter intake could be taken into account [34,35]. On the other hand, if data are collected from many more animals, more robust models could be set up in which for example interaction effects between herd, parity and DIM could also be considered to adapt the models of each trait individually to the physiological conditions.

The main limitation of this study is that the application of MPA to the sample data set cannot be validated because neither diagnoses of a veterinarian nor other subjective observations of the health status are available. Efforts should therefore be made to validate the MPA on a further data set against real diagnoses, ideally with extensive health data.

## 4. Conclusions

In this study, a concept for multivariate plausibility assessment of simultaneously recorded data was developed, in which individual observations were classified as ‘physiologically normal’, ‘implausible’ or ‘physiologically extreme’. The probabilistic approach, which is based on statistical models, aims to ensure adequate data quality so that the likelihood of distortions due to technical malfunctions is minimized for further investigations. The application to a hierarchically structured data set with 11 different traits showed the ability to identify most likely disease-related and thus physiologically extreme observations as in the example of clinical mastitis. Furthermore, observations of measurement boluses with non-physiological intra-reticular pH values were detected systematically. Even with such a complex data set as the one used here, where both the hierarchical data structure and different temporal resolutions in the data collection were challenging, the developed concept revealed to be a flexible method to improve the data quality for downstream analyses.

## Figures and Tables

**Table 1:** Descriptive statics of the data set before and after the applied multivariate plausibility assessment (MPA) ^1^.

Variable	Before MPA							After MPA						
	n_A_	n	Mean	SD	Min	Median	Max	n_A_	n	Mean	SD	Min	Median	Max
Climate														
Daily Mean Temperature (s.T), °C		160	12.1	6.2	−1.3	12.8	25.0		160	12.1	6.2	−1.3	12.8	25.0
Animal														
Lactation stage (DIM), d	100	1600	20.1	7.5	3	20	41	100	1600	20.1	7.5	3	20	41
Parity	100	1600	3.1	1.6	1	3	8	100	1600	3.1	1.6	1	3	8
Reticular measurement bolus														
Daily mean pH (r.pH)	93	1392	6.19	0.25	5.05	6.21	7.76	**89**	**1327**	**6.18**	**0.16**	**5.35**	6.21	**6.71**
Daily median Temperature (r.T_med_), °C	93	1392	39.37	0.36	38.30	39.30	41.30	93	**1380**	**39.36**	**0.34**	38.30	39.30	41.30
Noseband halter														
Rumination duration (Σ.Rt), h/d	97	1222	9.32	1.42	1.07	9.58	12.47	**95**	**1195**	**9.42**	**1.25**	1.07	**9.62**	12.47
Eating duration (Σ.Et), h/d	97	1222	5.37	1.39	1.40	5.37	12.45	**96**	**1209**	**5.31**	**1.27**	1.40	**5.36**	**9.90**
Pedometer														
Lying duration (Σ.Lt), h/d	98	1197	10.51	2.80	0.87	10.77	18.38	98	**1193**	**10.53**	**2.77**	0.87	10.77	18.38
Milk														
Milk yield (MY), kg/d	99	1126	37.5	8.5	5.2	38.0	63.8	99	1117	37.5	8.5	5.2	38.1	63.8
Somatic cell count (SCC), 1000/mL	99	365	310.7	974.2	6.5	60.2	13,067.4	99	**361**	**260.1**	**679.8**	6.5	**59.3**	**4901.6**
Somatic cell score ^2^ (SCS)	99	365	2.7	2.0	−0.9	2.3	10.0	99	**361**	**2.6**	**1.9**	−0.9	**2.2**	**8.6**
MIR spectrum	99	365						99	**357**					
Blood														
BHB, mmol/L	99	380	0.92	0.60	0.27	0.77	5.13	99	380	0.92	0.60	0.27	0.77	5.13
Log(BHB), log(mmol/L)	99	380	−0.09	0.21	−0.57	−0.12	0.71	99	380	−0.09	0.21	−0.57	−0.12	0.71
NEFA, mmol/L	99	379	0.38	0.24	0.01	0.33	1.89	99	**377**	0.38	0.24	**0.06**	0.33	1.89
Log(NEFA), log(mmol/L)	99	379	−0.50	0.28	−2.00	−0.48	0.28	99	**377**	**−0.49**	**0.26**	**−1.22**	−0.48	0.28
Glucose (GLU), mg/dl	99	381	57.33	9.76	8.90	57.20	147.10	99	**378**	**57.34**	**7.93**	**31.80**	57.20	**79.20**

^1^ BHB = β-hydroxybutyrate, DIM = days in milk, MIR = mid-infrared, n_A_ = number of animals, n = number of observations, NEFA = non-esterified fatty acids, SD = standard deviation. Numbers that have changed in the comparison before and after the MPA are written in bold. ^2^ The somatic cell score (SCS) was calculated according to Wiggans and Shook [20].
